# On the Importance of Including Cohesive Zone Models in Modelling Mixed-Mode Aneurysm Rupture

**DOI:** 10.1007/s13239-024-00740-3

**Published:** 2024-07-10

**Authors:** J. Concannon, E. Ó. Máirtín, B. FitzGibbon, N. Hynes, S. Sultan, J. P. McGarry

**Affiliations:** 1 Biomedical Engineering, University of Galway, Galway, Ireland; 2 Department of Vascular and Endovascular Surgery, Galway University Hospitals, Galway, Ireland

**Keywords:** Cohesive zone model, Aneurysm rupture, Finite element analysis, Delamination, Patient-specific

## Abstract

**Introduction:**

The precise mechanism of rupture in abdominal aortic aneurysms (AAAs) has not yet been uncovered. The phenomenological failure criterion of the coefficient of proportionality between von Mises stress and tissue strength does not account for any mechanistic foundation of tissue fracture. Experimental studies have shown that arterial failure is a stepwise process of fibrous delamination (mode II) and kinking (mode I) between layers. Such a mechanism has not previously been considered for AAA rupture.

**Methods:**

In the current study we consider both von Mises stress in the wall, in addition to interlayer tractions and delamination using cohesive zone models. Firstly, we present a parametric investigation of the influence of a range of AAA anatomical features on the likelihood of elevated interlayer traction and delamination.

**Results:**

We observe in several cases that the location of peak von Mises stress and tangential traction coincide. Our simulations also reveal however, that peak von Mises and intramural tractions are not coincident for aneurysms with Length/Radius less than 2 (short high-curvature aneurysms) and for aneurysms with symmetric intraluminal thrombus (ILT). For an aneurysm with (L/R = 2.0), the peak ${\sigma }_{vm}$ moves slightly towards the origin while the peak ${T}_{t}$ is near the peak bulge with a separation distance of ~ 17 mm. Additionally, we present three patient-specific AAA models derived directly from CT scans, which also illustrate that the location of von Mises stress does not correlate with the point of interlayer delamination.

**Conclusion:**

This study suggests that incorporating cohesive zone models into clinical based FE analyses may capture a greater proportion of ruptures in-silico.

**Supplementary Information:**

The online version contains supplementary material available at 10.1007/s13239-024-00740-3.

## Introduction

AAAs are deemed common in the general population with a global prevalence of 4.8%, and an 80% mortality rate in the event of rupture [1]. A number of finite element frameworks have been proposed in an attempt to improve AAA rupture risk estimates based on stress computations, however their ability to accurately predict rupture locations is just over 50% [2]. The maximum AAA diameter has widely been used to justify the need for surgical intervention, with AAAs greater than 5.5 cm in diameter deemed to require elective surgery [3]. However, clinical studies conducted by Darling et al. and Hall et al. determined that rupture rates of between 12 and 23% have been reported for AAAs less than 5 cm in diameter and rupture rates of 40% have been reported for AAAs greater than 5 cm [4, 5].

**Fig. 1:**
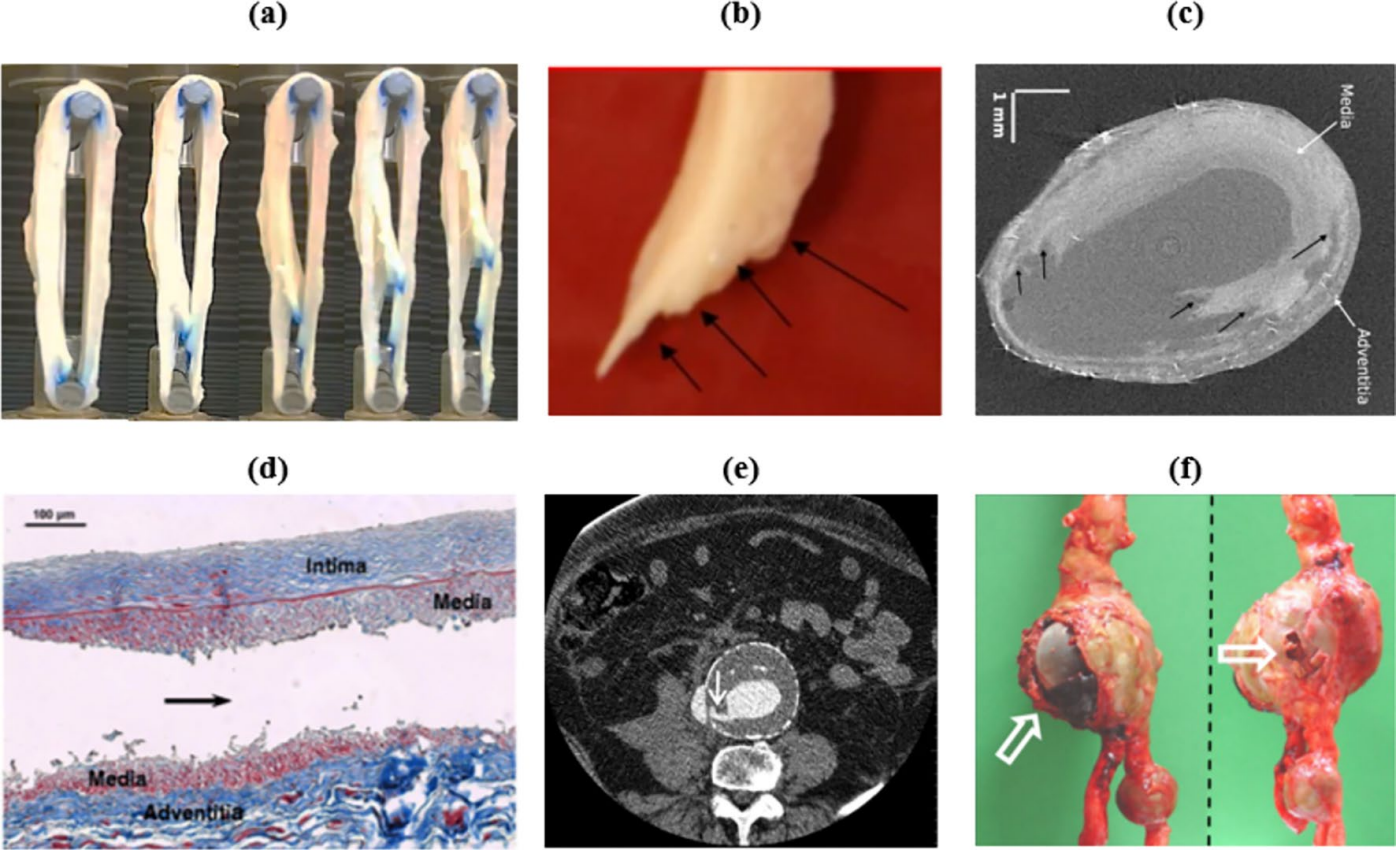
**a** Kinking intramural fracture of aortic tissue during ring pull test [8]. **b** Step rupture surface shows sequential separation between adjacent radial and then circumferential fascicles [20]. **c** Delamination planes at the rupture zone following tension-inflation [7]. **d** Histological evidence of jagged arterial delamination within the medial layer [21]. **e** Contrast Enhanced CT image of impending AAA rupture with stepped rupture surface [22]. **f** Complex failure of patient-specific AAA wall [23]

Accurate in-silico predictions of rupture potential indices rely on an in depth understanding of the process involved. Haslach et al. reported a stepped rupture surface following pressure inflation of rings of arterial tissue (Fig. 1b). It is generally accepted that medial degeneration is an essential prerequisite for interface delamination [6], which indeed may reduce the pressures required to induce a mixed mode-type failure. The existence of delamination planes at the rupture zone following tension-inflation inside an X-ray microtomography setup were reported by Brunet et al. [7] (Fig. 1c). FitzGibbon and McGarry [8] also report a step-wise interface delamination of aortic tissue when subject to a ring pull test (Fig. 1a). Dissection-like rupture was found in most burst-inflation samples by Romo et al. [9] who also report that rupture often initiated at a different location to the peak stress, while similar experiments by Kim et al. [10] revealed in certain cases that delamination occurred before AAA rupture. The jagged plateau region of load-extension plots performed by Pasta et al. [11] and Purslow [12] suggests that rupture does not propagate at a steady rate but arrests and reinitiates at somewhat regular intervals reflecting the fibrous nature of the wall. Altogether, these studies suggest that intramural delamination should be investigated as a potentially important factor in the process of AAA rupture.

Cohesive zone models (CZMs) have been used to predict delamination in various biological tissues previously, including bone [13], brain [14] and cells from substrates [15]. Clinical evidence has shown that maximal diameter was not significantly associated with rupture status [16], while a systematic review showed that aneurysmal wall characteristics (including intraluminal thrombus (ILT) and compliance) showed no association with AAA rupture [17]. Studies however, continue to use metrics such as equivalent strain to predict rupture [18], while Lorandon et al. [19] showed stress analysis in AAA does not predict rupture location correctly in patients with ILT.

In this study, interface delamination between the individual wall layers of the AAA is investigated using cohesive zone models (CZMs). Firstly, we present a parametric investigation of the influence of a range of AAA anatomical features on the likelihood of elevated interlayer traction and delamination. We observe in several cases that the location of peak ${\sigma }_{vm}$ and tangential traction (${T}_{t}$) coincide. Our simulations also reveal however, that peak ${\sigma }_{vm}$ and intramural tractions are not coincident for aneurysms with Length/Radius less than 2 (short high-curvature aneurysms) and for aneurysms with symmetric ILT. Additionally, we present three patient-specific AAA models derived directly from CT scans, which also illustrate that the location of ${\sigma }_{vm}$ does not correlate with the point of interlayer delamination.

## Methodology

In order to investigate the relationship between interface traction and ${\sigma }_{vm}$, a series of parametric investigations on the influence of a range of AAA anatomical features on the likelihood of elevated interlayer traction and delamination were performed, each of which were subject to the same internal pressure and boundary conditions. We then present three patient-specific aneurysm simulations derived directly from CT scans. The following subsections describe the idealised and patient-specific methodologies. In each case, Abaqus/Implicit is used and the CZM is implemented via a user-defined interface subroutine (UINTER) where traction-separation relationships (Eqs. 6a, 6b) and the Jacobian are defined. For further details on the CZM, the reader is referred to McGarry et al. [24]. A local cylindrical coordinate system prevents nodes at the proximal and distal neck of aneurysm from displacement in the axial direction and theta direction, such that said nodes only undergo radial deformation. A pressure load of 120 mmHg is applied to the internal lumen surface to mimic pulsatile conditions in-vivo due to blood pressure.

### Idealised Cases

**Fig. 2:**
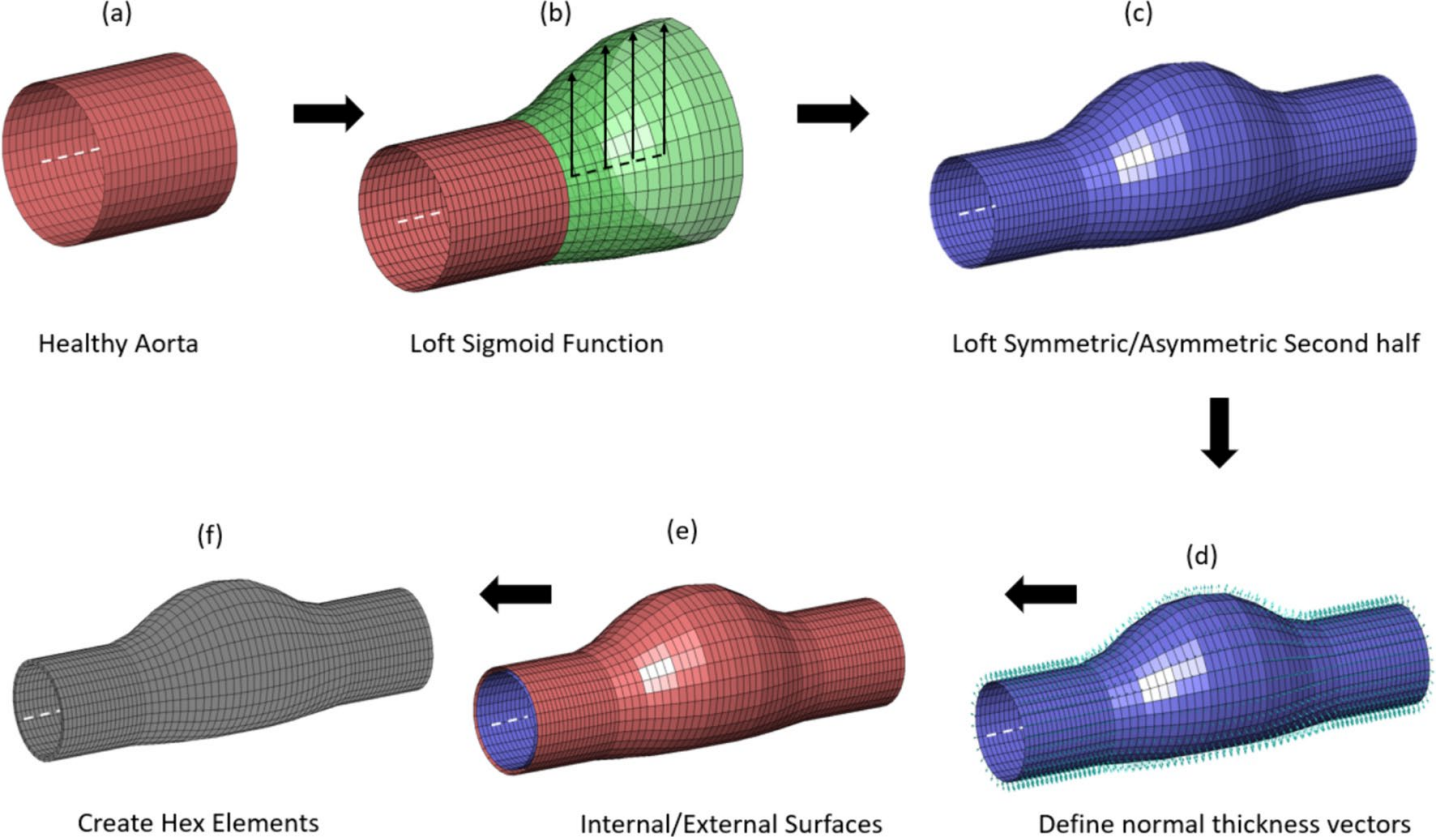
Flowchart describing idealised parametric study mesh creation. **a** A loft along the healthy proximal aortic segment to the point of the aneurysm neck. **b** A sigmoid function describes the aneurysm width as a function of the axial location. This is lofted onto the previously generated healthy aortic segment from (**a**). **c** The second half of the aneurysm mesh is created in a similar manner with allowable symmetric/asymmetric constraints. **d** The element normal thickness vectors are defined which allows creation of the external surface elements (**e**), and finally hexahedral elements are created between the two surfaces to define the final aneurysm mesh (**f**)

**Fig. 3:**
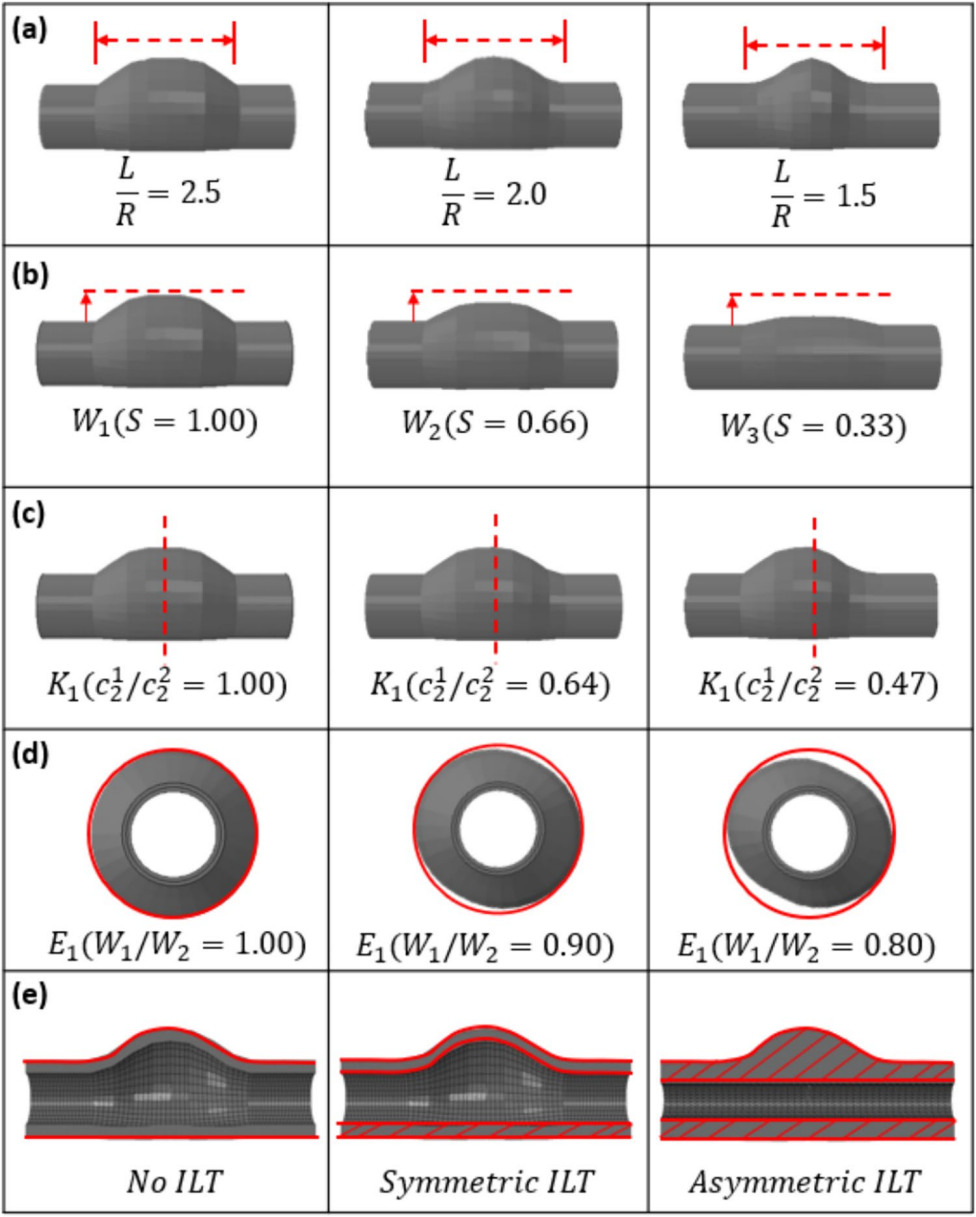
Idealised parametric study geometries created using Matlab. **a** Curvature (Length (L)/Radius (R))—where R is fixed at 20 mm and L = [50 mm, 40 mm, and 30 mm], **b** Width (W_1_) where W_1_ = R*S, (R = 20 mm, S = [1.00, 0.66, 0.33]). **c** Skewness, K(C_2_^1^/C_2_^2^), where C_2_^1^ = 7 and C_2_^1^ = [7, 11, 15]. **d** Ellipticity E(W_1_/W_2_), where W_1_ is as previously defined above and W_2_ = [1.00, 0.90, 0.80]. **e** ILT (symmetric/asymmetric)

A series of finite element models were created using MATLAB (R2017b, MathWorks) based on a sigmoid function

1 $${\Theta }_{r, x}=\frac{1}{1+{e}^{-{c}_{1}\left(x-{c}_{2}\right)}}$$

where ${\Theta }_{r, x}$ is the radial coordinate of the finite element mesh at the node corresponding to the axial coordinate, $x$, (Fig. 2). By altering the parameters ${c}_{1}$ and ${c}_{2}$ of the sigmoid function we investigate the effect of the following conditions on the interface traction/${{\varvec{\sigma}}}_{{\varvec{v}}{\varvec{m}}}$ relationship; (i) aneurysm curvature, (ii) aneurysm width, (iii) aneurysm skewness, (iv) aneurysm ellipticity, (v) symmetric and asymmetric ILT, and (vi) vessel anisotropy (Fig. 3). For brevity, results for skewness and ellipticity are shown in the Supplementary Material.

The following cases were investigated; (a) Curvature (L/R)—where R (radius) is fixed at 20 mm and L (length) = [50 mm, 40 mm, and 30 mm] defined by the c_2_ parameter in the sigmoid function, (b) Width (W_1_) where W_1_ = R*S, (R = 20 mm, S = [1.00, 0.66, 0.33]) and is implemented through a radial shrinkage of the lumen coordinates by the factor S prior to pressure inflation. (c) Skewness (C_2_^1^/C_2_^2^), where C_2_^1^ = 7 and C_2_^2^ = [7, 11, 15] which defines the degree of axial asymmetry along the aneurysm length. (d) Ellipticity (W_1_/W_2_), where W_1_ is as previously defined above and W_2_ = [1.00, 0.90, 0.80], which is implemented through a circumferentially descending multiplicator that is applied to the lumen coordinates to achieve varying degrees of aneurysm ellipticity. (e) ILT (symmetric/asymmetric), symmetric ILT is simply achieved by offsetting the internal lumen coordinates inwards by the symmetric ILT thickness t_1_. Asymmetric ILT is achieved by defining the internal lumen and filling the void between the lumen surface and the internal wall surface with tetrahedral elements using GIBBON [25]. For simplicity, each of the parameters associated with the idealised parameteric analyses along with their descriptions are shown in the Supplementary Materials.

#### Vessel Anisotropy

Material anisotropy is considered using a bilinear-type formulation [26] where the strain energy function is given as

2 $${\Psi }_{\text{tot}}{={\Psi }_{\text{iso}}+\Psi }_{aniso}$$

3 $${\Psi }_{aniso}={\sum }_{i=\text{1,2}}\left\{\begin{array}{cc}{E}_{1f}\left(\frac{2}{3}{\lambda }_{fi}^{3}-{\lambda }_{fi}^{2}+\frac{1}{3}\right)& {\lambda }_{fi}-1\le {D}_{1f}\\ \frac{2}{3}{\lambda }_{fi}^{3}\left({q}_{f}-2{p}_{f}\right)+\frac{{p}_{f}}{2}{\lambda }_{fi}^{4}+{\lambda }_{fi}^{2}\left({p}_{f}-{q}_{f}+{r}_{f}\right)+{\psi }_{01}& {D}_{1f}<{\lambda }_{fi}-1<{D}_{2f}\\ \frac{2{E}_{2f}}{3}{\lambda }_{fi}^{3}+{\lambda }_{fi}^{2}\left({p}_{f}{D}_{2f}^{2}+{q}_{f}{D}_{2f}+{r}_{f}-{E}_{2f}-{E}_{2f}{D}_{2f}\right)+{\psi }_{02}& {\lambda }_{fi}-1 \ge {D}_{2f}\end{array}\right.$$

where

4a $${p}_{f}={(E}_{1f}-{E}_{2f})/{2(D}_{1f}-{D}_{2f})$$

4b $${q}_{f}={E}_{1f}-2{D}_{1f}{p}_{f}$$

4c $${r}_{f}=\left({E}_{1f}-{q}_{f}\right){D}_{1f}-{p}_{f}{D}_{1f}^{2}$$

and ${\psi }_{01}$ and ${\psi }_{01}$ are two constants which ensure the continuity of strain energy. ${E}_{1f}$ and ${E}_{2f}$ are slopes of the linear regimes, ${D}_{1f}$ and ${D}_{2f}$ are the values of the nominal fibre strain at the end of the first linear regime and at the beginning of the second linear regime. The bilinear strain-stiffening fibre model is placed in parallel with a linear neo-Hookean matrix

5 $${\Psi }_{iso}\left(\mathbf{C}\right)=\frac{K}{2}{\left(J-1\right)}^{2}+\frac{\mu }{2}({\overline{I} }_{1}-3)$$

where $K$ is the effective bulk modulus, $\mu$ is the effective shear modulus, and ${\overline{I} }_{1}$ is the first invariant of the isochoric right Cauchy-Green Tensor ($\overline{\mathbf{C} }={J}^{-\frac{2}{3}}\mathbf{C}$).

**Fig. 4:**
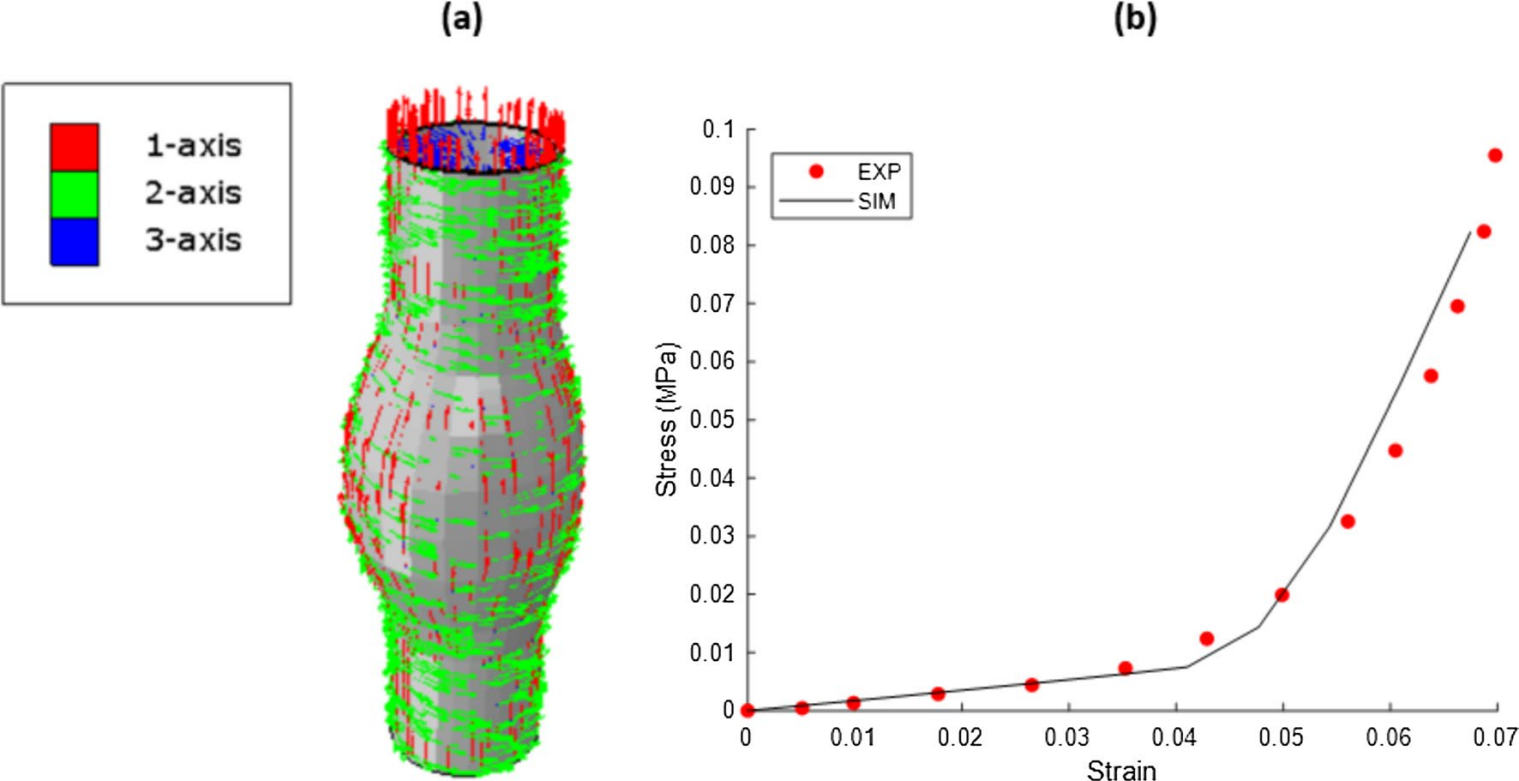
**a** Example of discrete fiber orientation applied to aneurysm model. Collagen fibers are defined with respect to each individual element orientation and prescribed via the anisotropic material formulation such that the material captures the stress-strain response to AAA uniaxial tension experiments. **b** Experimental tension experiments of Vande Geest et al. [27] (EXP) with finite element model result overlaid (SIM)

Figure 4 below shows the simulated stress-strain response (SIM) with the experimental data of Vande Geest et al. [27] overlaid.

The interface between each artery wall layer is modelled using the non-potential-based SMC and NP2 CZMs presented in [24], (SMC model is used in separation and NP2 model is used in compression/over-closure),

6a $${T}_{n}\left({\Delta }_{n},{\Delta }_{t} \right)={\sigma }_{max}exp\left(1\right)\left(\frac{{\Delta }_{n}}{{\delta }_{n}}\right) exp\left(-\sqrt{\frac{{\Delta }_{n}^{2}}{{\delta }_{n}^{2}}+\frac{{\Delta }_{t}^{2}}{{\delta }_{t}^{2}}}\right)$$

6b $${T}_{t}\left({\Delta }_{n},{\Delta }_{t} \right)={\tau }_{max}exp\left(1\right)\left(\frac{{\Delta }_{t}}{{\delta }_{t}}\right) exp\left(-\sqrt{\frac{{\Delta }_{n}^{2}}{{\delta }_{n}^{2}}+\frac{{\Delta }_{t}^{2}}{{\delta }_{t}^{2}}}\right)$$

where ${T}_{n}$ is the traction in the normal direction;${T}_{t}$ is the traction in the tangential direction; ${\Delta }_{n}$ is the normal component of the interface separation vector; ${\Delta }_{t}$ is the tangential component of the interface separation vector; ${\sigma }_{max}$ is the normal interface strength; ${\tau }_{max}$ is the tangential interface strength; ${\delta }_{t}$ is the tangential interface characteristic length; and ${\delta }_{n}$ is the normal interface characteristic length. Values of maximum interface strengths in the normal $\left({\sigma }_{max}\right)$ and tangential $\left({\tau }_{max}\right)$ directions are based on experiments of human aortic tissue following autopsy [28]. Specifically, an interaction strength of ${\sigma }_{max}={\tau }_{max}=16kPa$ and a critical cohesive interface length of ${\delta }_{c}=10\mu m$ is chosen.

### Patient-Specific Cases

**Fig. 5:**
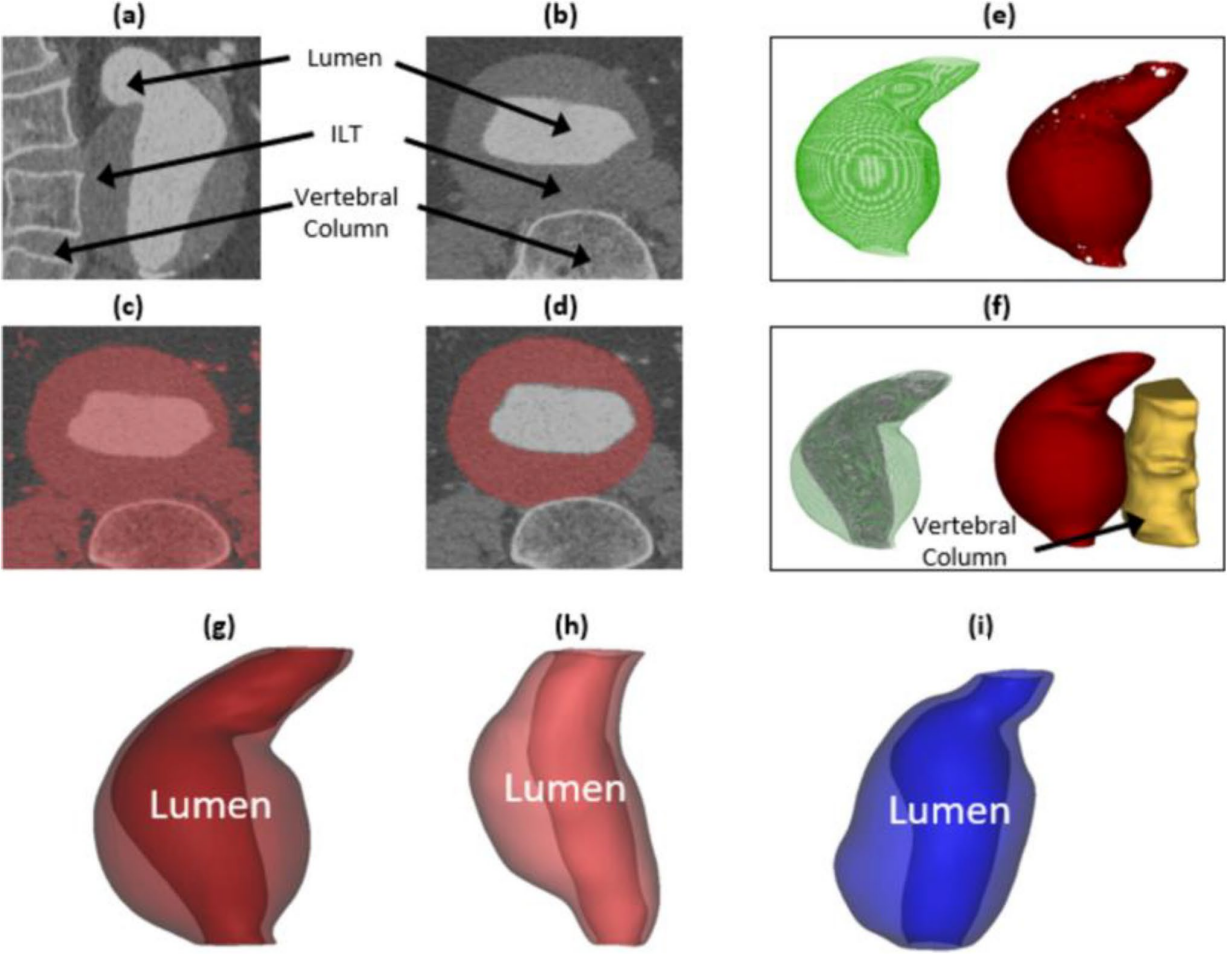
Outline of the reconstruction steps taken to create final AAA geometry. CT scans of the aneurysm (Patient A) in the sagittal and transverse planes are shown in (**a**) and (**b**) respectively. The initial and final aneurysm ‘mask’ in the transverse plane is shown in (**c**) and (**d**) respectively. The initial and final 3D AAA geometry together with surface polylines is shown in (**e**) in (**f**) respectively with the position of the vertebral column also indicated. **g**–**i** indicate final AAA geometries (1, 2, 3) for three clinical cases

Patient-specific CT scans were obtained with permission from the Western Vascular Institute, University College Hospital, Galway following project approval by the hospital ethics committee. Digital imaging and communication in medicine (DICOM) files containing the CT data were imported into Mimics^©^ (v14.11, Materialise, Belgium) for segmentation and reconstruction. An illustration summarising the reconstruction process, from CT scans to final geometry, is shown in Fig. 5. The final reconstructed 3D geometry of the ILT (for Patient A), including the vertebral column, is shown in Fig. 5f. In total, three AAA geometries, shown in Fig. 5g–i, were analysed. For the remainder of this study these AAA geometries will be referred to as Patient A, Patient B and Patient C.

After importing the ILT geometry into Abaqus, intima, media and adventitia layers were generated by offsetting the geometry from the abluminal surface of the ILT. In each of the idealised and patient-specific cases the individual layer thicknesses are based on those reported in Concannon et al. [29]. A hypertensive pressure of 200 mmHg is applied to simulate blood pressure acting on the AAA wall, in order to allow for analysis of delamination pattern spreading (e.g. Fig. 11). The AAA geometry is partially constrained in the proximal and distal regions, representing the crux of the diaphragm and the common iliac bifurcation in-vivo. A tie constraint is applied between the aneurysm and the vertebral column surfaces.

## Results

We first investigate a suite of parameterised geometries that capture the typical spectrum of AAAs which present clinically and observe in most cases that the location of peak ${\sigma }_{vm}$ and ${T}_{t}$ coincide. There are, however, certain cases where the locations do not coincide, which suggests that a cohesive zone model should be used in addition to ${\sigma }_{vm}$ in finite element-based rupture risk models. Additionally, we present three patient-specific AAA models derived directly from CT scans, which also illustrate that the location of peak ${\sigma }_{vm}$ does not correlate with the point of interlayer delamination. Kleinloog et al. [30] found curvature to be an inconsistent indicator of rupture, in a systematic review of 102 studies, while Lane et al. [31] demonstrated a dependence of curvature on rupture in a murine aneurysm model. Hall et al. [5] described the relationship between aortic wall stress predicted using the Law of Laplace (i.e., based on maximum AAA diameter) and risk of AAA rupture, however in-vitro testing on aneurysms show that rupture location doesn’t coincide with the maximum radius location [9]. Therefore it was deemed essential to perform a parametric analysis on the affect of length/radius ratio on von mises stress and interface tractions.

### Idealised Cases

#### Aneurysm Curvature (L/R)

**Fig. 6:**
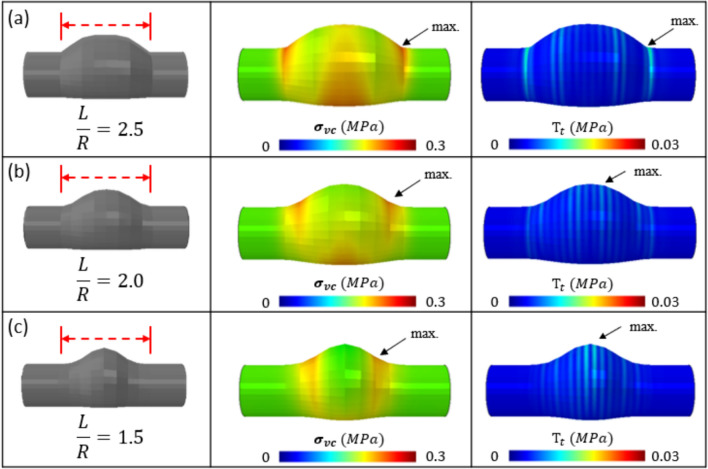
Effect of Curvature (L/R) on the peak ${\sigma }_{vm}$/${T}_{t}$ relationship. **a** Baseline geometry (L/R = 2.5): peak ${\sigma }_{vm}$ and ${T}_{t}$ are coincident at the aneurysm neck. **b** L/R = 2.0: peak ${\sigma }_{vm}$ and ${T}_{t}$ are not-coincident, and **c** L/R = 1.5: peak ${\sigma }_{vm}$ and ${T}_{t}$ are not-coincident

**Fig. 7:**
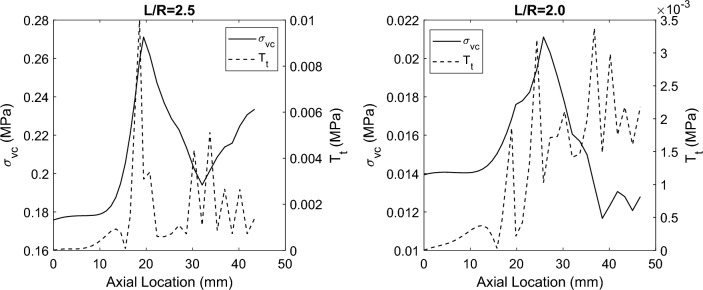
Distribution of von Mises stress and tangential tractions along the aneurysm axial dimension. Axial Location = 0 mm indicates the proximal normal aorta, ~ 20 mm indicates the beginning of the aneurysm, and ~ 45 indicates the maximal diameter. (Left): For L/R = 2.5, the location of peak Von Mises stress and tangential tractions are coincident at the mouth of the aneurysm. (Right) For L/R = 2.0, the peaks are separated by ~ 11 mm. In all simulations, computed normal interface tractions are negligible, indicating that interlayer dissection is primarily mode II in nature

**Fig. 8:**
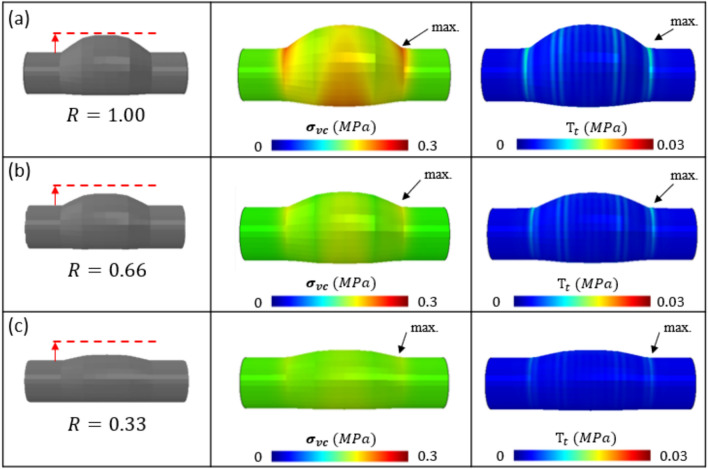
Effect of Width (${W}_{1}$) on the peak ${\sigma }_{vm}$/${T}_{t}$ relationship. **a** Baseline geometry (${W}_{1}=1.00$), **b** ${W}_{1}=0.66$, and **c** ${W}_{1}=0.33$. In each case the peak ${\sigma }_{vm}$ and ${T}_{t}$ are coincident at the aneurysm neck, and L = 50mm

**Fig. 9:**
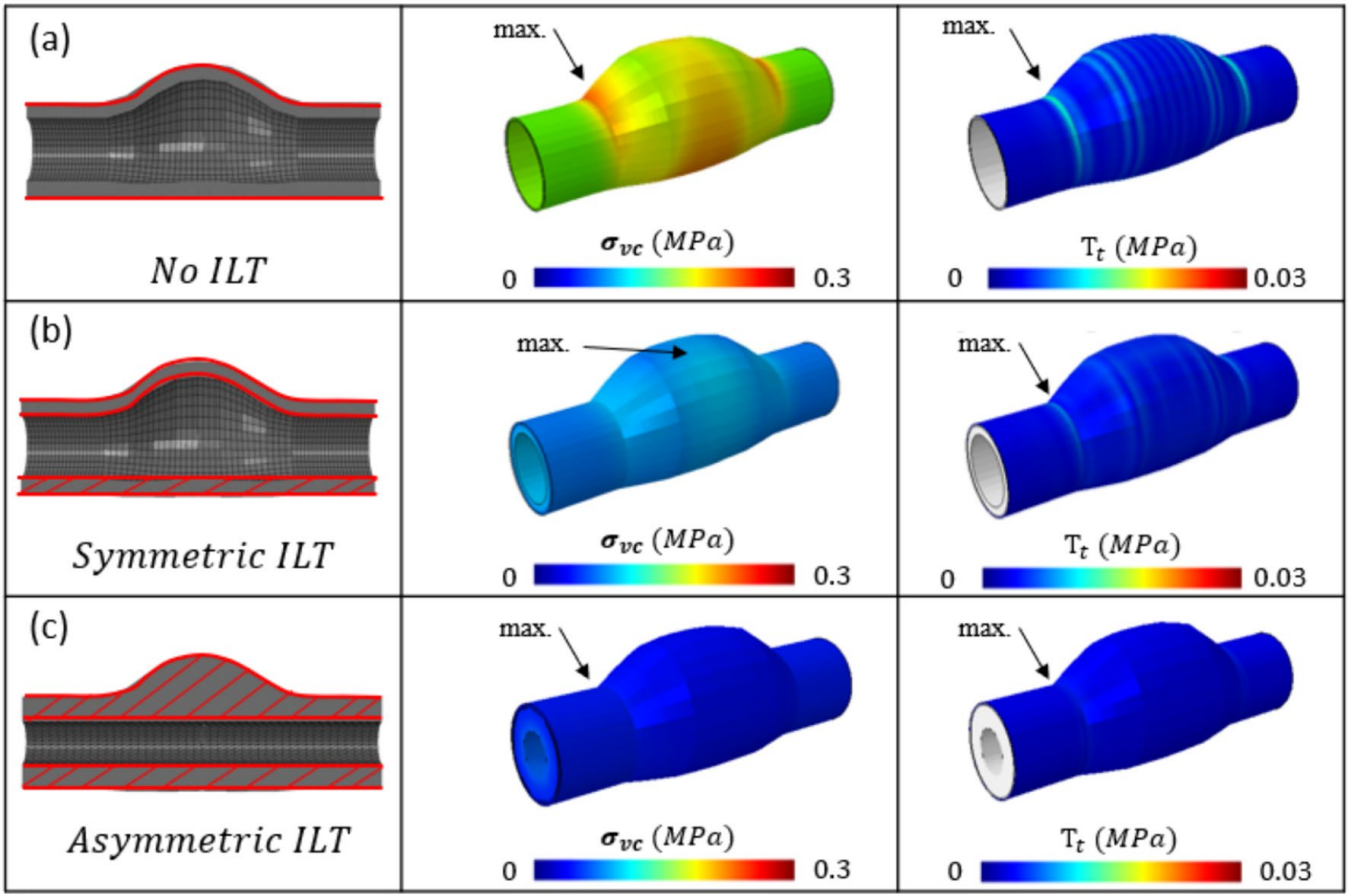
Effect of ILT on the peak ${\sigma }_{vm}$/${T}_{t}$ relationship. **a** Baseline geometry (No ILT), **b** Symmetric ILT, and **c** Asymmetric ILT. In the case of symmetric ILT, the peak ${\sigma }_{vm}$ and ${T}_{t}$ do not coincide, however in the case of asymmetric ILT they do at the aneurysm neck

The baseline geometry in each case is presented in the first row of Figs. 6, 8, 9, and consists of an anteriorly distending AAA, with a width (R) (in-plane distance from central axis to anterior bulge) of 20 mm, axial length (L) of 50 mm, wall thickness of 1 mm, and healthy proximal and distal segments of 10 mm radius and 20 mm length. Figure 6 illustrates the effects of altering the curvature (L/R) of the aneurysm on the ${\sigma }_{vm}$/${T}_{t}$ relationship. In the baseline geometry (L/R = 2.5), the peak ${\sigma }_{vm}$/${T}_{t}$ locations are coincident, at the aneurysm neck. For an aneurysm with (L/R = 2.0), the peak ${\sigma }_{vm}$ moves slightly towards the origin while the peak ${T}_{t}$ is near the peak bulge with a separation distance of ~ 17 mm. Similarly, for an aneurysm with (L/R = 1.5), the peak ${\sigma }_{vm}$ moves slightly closer again to the origin, while the peak ${T}_{t}$ is near the peak bulge with a separation distance of ~ 22 mm (Fig. 7).

#### Aneurysm Width (${\text{W}}_{1}$)

Next, we investigated the effect of varying the aneurysm width on the peak $${\sigma }_{vm}$$/$${T}_{t}$$ relationship. In each case ($${W}_{1}=1.00, 0.66, 0.33)$$, although the peak $${\sigma }_{vm}$$ and $${T}_{t}$$ values reduce with decreasing aneurysm width, they remain coincident at the aneurysm neck (Fig. 8).

#### Intraluminal Thrombus (ILT)

Here we include both symmetric and asymmetric ILT in our parametric study. In the symmetric ILT case, the peak ${\sigma }_{vm}$ and ${T}_{t}$ do not coincide, with the ${\sigma }_{vm}$ maximum at the aneurysm belly (point of maximum radius) while the ${T}_{t}$ resides at the aneurysm neck (point at which healthy aorta and aneurysm meet). In the asymmetric ILT case, the peak ${\sigma }_{vm}$ and ${T}_{t}$ do coincide at the aneurysm neck. In each case here, L/R = 2.5, and C_2_^1^/C_2_^2^ = 1.00 (Fig. 9).

### Patient-Specific Cases

#### Patient A

**Fig. 10:**
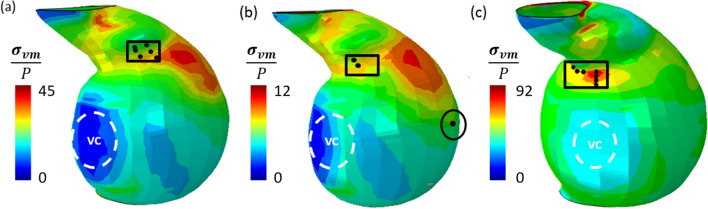
Distribution of computed von Mises stress over pressure in **a** the adventitia layer and **b** the media layer at an applied lumen pressure (P) of 150 mmHg for Patient A. **c** indicates the distribution of computed von Mises stress over pressure in the adventitia layer at an applied lumen pressure (P) of 93 mmHg for Patient A where the intraluminal thrombus (ILT) is omitted. The position of the vertebral column (VC) is also indicated

In Fig. 10 contour plots of the ${\sigma }_{vm}$**/**P distribution in the adventitia and media layer are presented, with a selection of nodes in the computed interface delamination patches highlighted for comparison of location (black circles). It is evident that the predicted location of interface delamination at the adventitial-medial (A-M) interface does not coincide directly with the location of maximum ${\sigma }_{vm}$ in the adventitia layer. Furthermore, predicted interface delamination at the intimal-medial (I-M) interface do not coincide with the peak ${\sigma }_{vm}$ location in the media layer. In particular, the computed delamination location in the anterior region (circled) at the I-M interface coincides with a region in which the computed magnitude of ${\sigma }_{vm}$ in the media layer is relatively low. In contrast, when the ILT is neglected, predicted interface delamination locations at the adventitia-media interface roughly coincide with the peak ${\sigma }_{vm}$**/**P region in the adventitia layer.

#### Patient B

**Fig. 11:**
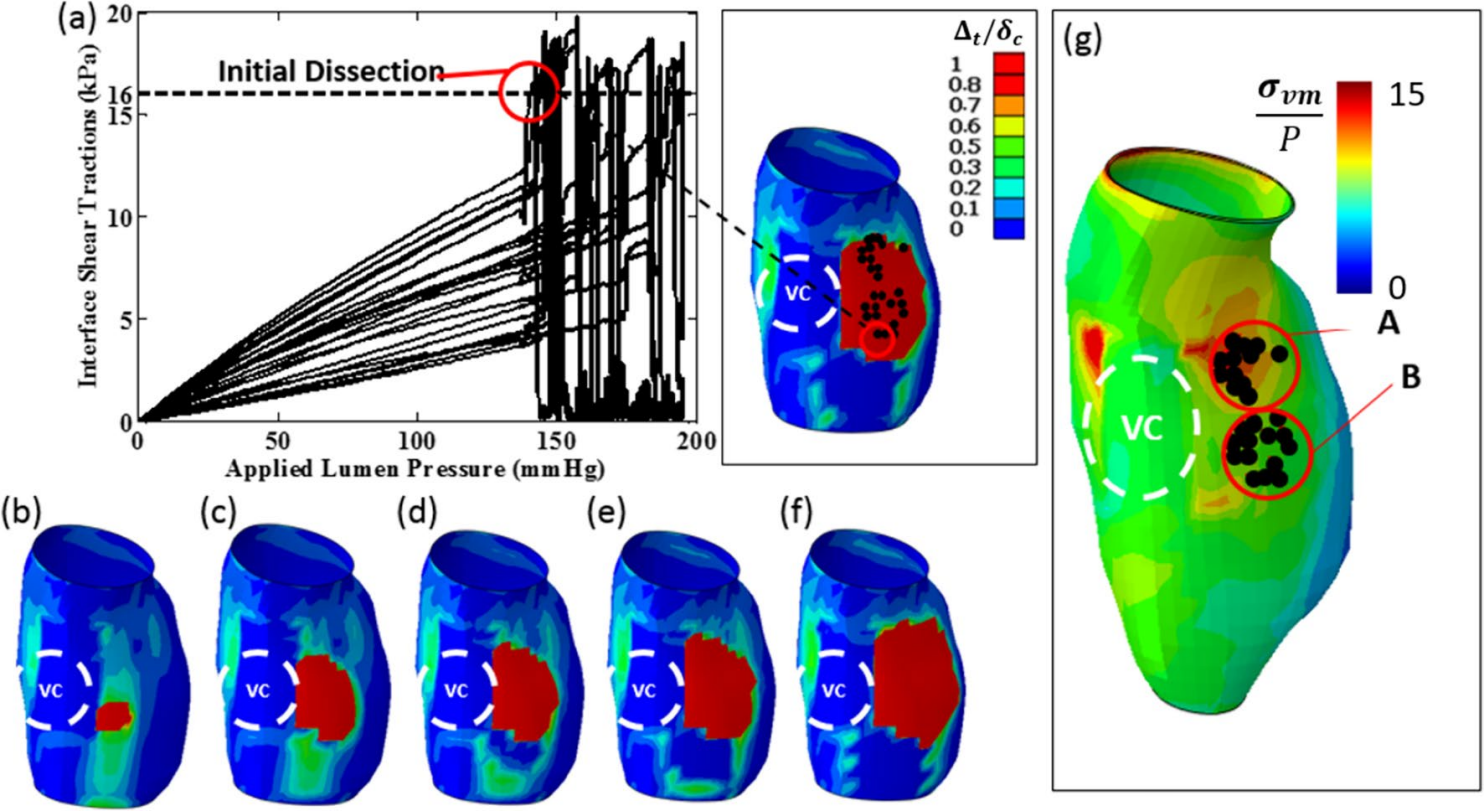
**a** Computed interface shear traction as a function of applied lumen pressure at the intima-media interface for Patient B. A significant region in which the critical cohesive characteristic distance is exceeded $({\Delta }_{t}/{\delta }_{c}>1)$ is shown. The initial computed delamination region is also indicated. The delamination patch is also shown for a lumen pressure of: **b** 142 mmHg; **c** 160 mmHg; **d** 170 mmHg; **e** 180 mmHg and **f** 195 mmHg. **g** Distribution of computed ${\sigma }_{vm}$/P in the media layer at an applied lumen pressure (P) of 142mmHg

A significant region of delamination is predicted at the I-M interface to the right of the vertebral column (VC) as shown in Fig. 11. The associated interface shear traction evolution for a selection of nodes in the delamination patch are also depicted. The progression of the interlayer crack front is shown in Fig. 11b–f as the lumen pressure is increased from 142 to 195 mmHg. The computed ${\sigma }_{vm}$**/**P distribution in the media layer (when the ILT is included) is presented in Fig. 11g. A selection of nodes in the delamination patch are superimposed for comparison. The initial delamination region, highlighted by circle ‘B’, does not correlate with a significant ${\sigma }_{vm}$**/**P concentration.

#### Patient C

**Fig. 12:**
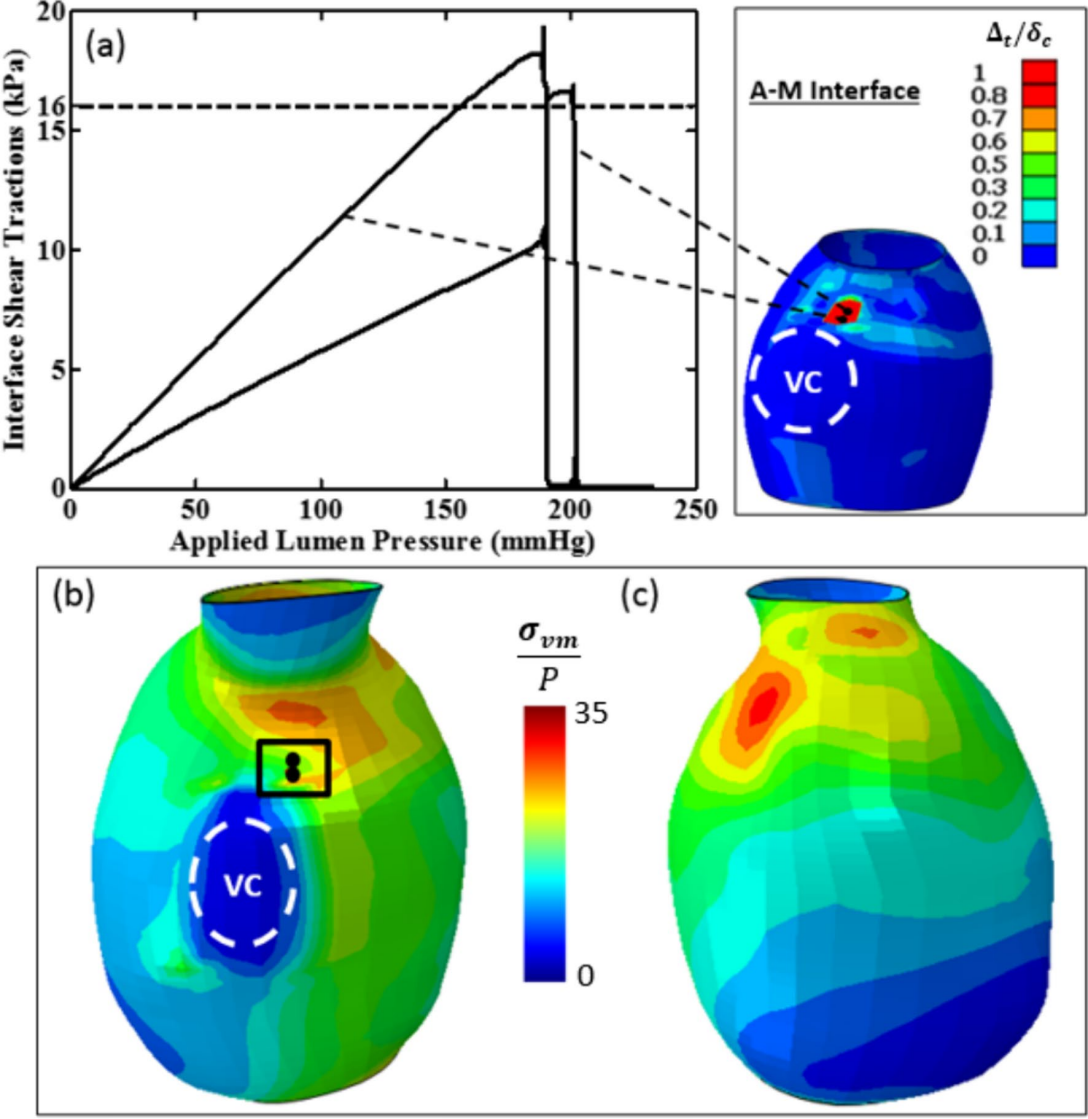
**a** Computed interface shear traction as a function of applied lumen pressure at the adventitia-media interface for Patient C. A significant region in which the critical cohesive characteristic distance is exceeded $({\Delta }_{t}/{\delta }_{c}>1)$ is shown. **b**, **c** posterior and anterior distribution of computed ${\sigma }_{vm}$/P in the adventitia layer at an applied lumen pressure of 188 mmHg for Patient C where the intraluminal thrombus (ILT) is included

A region of delamination is predicted at the A-M interface just above the position of the vertebral column (VC) as shown in Fig. 12. The associated shear traction evolution for two nodes in the delamination patch is also depicted. Initial interface delamination is computed at 188 mmHg. Following complete interlayer delamination at this node, characterised by a rapid decrease in interface traction, delamination is computed in an adjacent node in the delamination patch at 190 mmHg. No interlayer delamination is computed at the I-M interface. The computed ${\sigma }_{vm}$/P distribution in the adventitia layer is presented in Fig. 12b and c for the posterior and anterior AAA sides respectively with the nodes in the delamination patch superimposed for comparison. It is evident that ${\sigma }_{vm}$**/**P concentrations do not coincide with predicted computed delamination locations.

### Comparison of Three AAA Geometries

A significant region of dissection is predicted at the intima-media interface to the right of the vertebral column (VC) is shown for Patient B. Following initial interface dissection, in the bottom left quadrant of the dissection patch, the interlayer crack front propagates towards the top right quadrant of the dissection patch following an increase in lumen pressure. The computed region of intima-media dissection is very different to that computed for patient A, where dissections of the intima-media interface occurs in the region of the aneurysm neck and in the anterior region. Additionally a lower rate of crack front propagation is predicted with increasing lumen pressure for Patient A. Interlayer dissection is also computed at the adventitia-media interface, initiating at approximately 150 mmHg. For Patient C, Additionally, it is worth noting that a localised region of increased von Mises stress is computed on the anterior AAA side. However, no interlayer dissection is computed in this region. initial interlayer dissection is computed at the adventitia-media interface for Patient C at an increased lumen pressure of 188 mmHg, with no dissection computed at the intima-media interface. The pronounced differences between the dissection predictions, in terms of initiation pressure, location and crack front propagation highlight the critical importance of geometric variation of AAA.

## Discussion

A key finding of the present study is that the location of delamination initiation does not always coincide with locations of peak ${\sigma }_{vm}$ in the artery wall, which suggests that further measures in addition to ${\sigma }_{vm}$, including interface shear strength should be included to improve rupture potential predictions for AAAs. Previous studies have modelled the AAA wall as a single homogenous entity, using the peak computed ${\sigma }_{vm}$ as a predictor for rupture, however, the specific rupturing mechanism of the tissue has not been considered in such formulations. Several experimental studies have reported jagged, stepwise mixed-mode failure surfaces of arteries, indicating that interlayer dissection represents a critical step in the rupture process of AAA tissue and should be considered in computational based RPIs.

Firstly, a parametric investigation is performed on a suite of aneurysm geometries that capture the typical spectrum of AAAs which present clinically. In several cases the location of peak ${\sigma }_{vm}$ and ${T}_{t}$ coincide, however in a select few there are significant discrepancies between peak locations. Where aneurysm curvature is concerned, a high L/R ratio of 2.5 results in coincident peaks in ${\sigma }_{vm}$ and ${T}_{t}$ at the aneurysm neck, as the L/R ratio decreases however, the locations of peak ${\sigma }_{vm}$ and ${T}_{t}$ become non-coincident. The width of the aneurysm, (W_1_), has no effect on the location of peak ${\sigma }_{vm}$ nor ${T}_{t}$, only their magnitudes, and they remain coincident as the value of W_1_ decreases. For a symmetric ILT, the location of peak ${\sigma }_{vm}$ is non-coincident with the peak ${T}_{t}$ location, whereas for an asymmetric ILT, their locations are coincident at the aneurysm neck.

In the second part of this study interlayer delamination is simulated for three AAA geometries reconstructed from patient-specific CT scans. In Patient A delamination initiates at the A-M interface with initial delamination computed at a lumen pressure of 132 mmHg. Delamination at the I-M interface initiates at a lumen pressure of 138 mmHg. These predictions of delamination initiation are supported by the study of [32] where AAA rupture is reported at a lumen pressure of 142 mmHg, indicating that the assumed values of interface strength are in the correct range for AAA tissue. Interface strengths of up to 140 kPa [33, 34] however have been reported for healthy human tissue, as evident in the fact that healthy tissue does not tend to dissect at pressures as low as 140 mmHg. Simulations for Patient B predict delamination initiation at a lumen pressure of 142 mmHg, again identical to the rupture pressure reported by [32]. However, the delamination location is very different to that computed for Patient A. In the case of Patient B, delamination initiates in the I-M layer, on the lateral right side of the aneurysm far from the neck region. While delamination initiates at a higher lumen pressure for Patient B, the crack front propagates at a much higher rate than that computed for Patient A, so that a very large delamination patch exists at a lumen pressure of 200 mmHg. Finally, initial interlayer delamination is computed at the A-M interface for Patient C at an increased lumen pressure of 188 mmHg, with no delamination computed at the I-M interface. The pronounced differences between the delamination predictions, in terms of initiation pressure, location and crack front propagation highlight the critical importance of understanding geometric variation of AAAs.

In addition to the importance of modelling the AAA wall as an inhomogeneous structure, this study also demonstrates the pronounced effect of the ILT on interlayer shear tractions. The presence of the ILT significantly alters the location of interlayer shear traction concentrations in comparison to simulations in which the ILT is excluded. Interestingly, when the ILT was removed from the three patient-specific geometries used in this study, rupture (as indicated by the CZM) initiated at a lower lumen pressure in all cases relative to simulations in which the ILT was included. The effect of the ILT on AAA wall stress has been reported previously [35, 36]. The present study furthers emphasises the importance of the ILT in terms of interlayer tractions and delamination prediction in an inhomogeneous wall.

The finding of the present study which shows that delamination locations do not always coincide with locations of ${\sigma }_{vm}$ concentrations is broadly supported by the study of Georgakarakos et al., in which it has been reported that aortic blebs, associated with increased risk of rupture, do not correlate with peak ${\sigma }_{vm}$ computed for a homogeneous AAA wall [37]. Additionally, Romo et al. [9] report that rupture locations were not coincident with locations of maximum stress following pressure inflation of thoracic aortic aneurysms. Intimal flaps and false lumens have been reported in the clinical literature for aneurysms, again suggesting that interlayer dissection is an important mode of damage, potentially contributing to total rupture [38]. Moreover, considering that interface strengths of 140 kPa [33] and 202 kPa [8] have been reported for healthy tissue and that the crack propagation length of aneurysmal tissue is ~ 3 times that of healthy tissue [39], it is certainly plausible that some factor governing interface strength is compromised in aneurysmal tissue. This is supported by our simulations which reveal that delamination can precede the critical ${\sigma }_{vm}$ required to cause rupture using the RPI criterion, highlighting the importance of the inclusion of cohesive zone models into computational based rupture prediction frameworks.

The results presented in this study suggest that if aneurysms grow uniformly that the location of peak von mises stress and peak interface tractions, remain coincident as the aneurysm grows. This has impotant implications for vascular surgeons and interventional radiologists regarding the decision to operate or not, as focus can be directed to the coincident point on the aneurysm. If however, we take the morphological features of Fig. 6c as the reference configuration, and this aneurysm grows longitudinally, to that presented in Fig. 6b, at low L/R ratios, locations are non-coincident, and surgeons should investigate both of these positions as potential areas of rupture. Moreover, if ILT appears between one follow up scan and the next, as indicated in Fig. 11, the locations become noncoincident, and both should be monitored.

## Conclusion

In conclusion, this study shows that delamination locations are not always found to correlate with locations of ${\sigma }_{vm}$ concentrations, suggesting that ${\sigma }_{vm}$ in the AAA wall should not be relied upon as the sole mechanical indicator of rupture risk, particularly given the experimental evidence of a mixed-mode rupture surface. Furthermore, initial interlayer delamination pressures and locations, in addition to the rate of delamination propagation are found to be highly influenced by the geometric variations. With current AAA rupture risk frameworks only predicting rupture in 53.8% of cases, the addition of cohesive zone models into clinical based FE analyses may improve their accuracy and provide additional information to surgeons in terms of whether the risk of rupture outweighs the risk of surgery for patients.

## Supplementary Information

Below is the link to the electronic supplementary material.

Supplementary file1 (DOCX 892 kb)
