# Supplementary material for: On the Importance of Including Cohesive Zone Models in Modelling Mixed-Mode Aneurysm Rupture
Source: Cardiovasc Eng Technol. 2024 Jul 10;15(5):633–46. doi: 10.1007/s13239-024-00740-3 (PMC11582104; doi:10.1007/s13239-024-00740-3)

**Table S1: decription of parameters relevant to creation of idealised finite element models in Figure 3.**

| **Parameter** | **Description** |
| --- | --- |
| L | Aneurysm length |
| R | Aneurysm radius |
| L/R | Aneurysm curvature |
| S | Radial node scale factor |
| $W$ | Aneurysm width |
| K | Aneurysm skewness (K=1 indicates symmetric about transverse plane) |
| E | Aneurysm ellipticity (E=1 indicates axisymmetric aneurysm) |
| ILT | Intraluminal Thrombus (none, symmetric, asymmetric) |

*Aneurysm Skewness* $(C_{2,1}/C_{2,2})$*:*

By altering the ratio of the $C_{2,1}$ to $C_{2,2}$ parameters in the sigmoid function, the skewness of the aneurysm can be altered. In each case ($C_{2,1}/C_{2,2}=1.00, 0.64, 0.47)$ the peak $\sigma_{vm}$ and $T_{t}$are coincident however their location follows the tighter radius of curvature at the aneurysm neck (**Figure S1**).


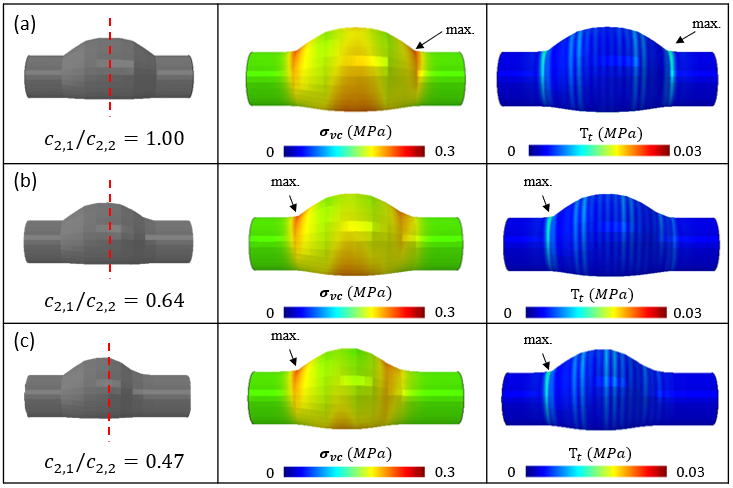


**Figure S1: Effect of Skewness (**$\boldsymbol{C}_{\boldsymbol{2,1}}\boldsymbol{/}\boldsymbol{C}_{\boldsymbol{2,2}}$**) on the peak** $\boldsymbol{\sigma}_{\boldsymbol{vm}}$**/**$T_{t}$ **relationship. (a) Baseline geometry (**$\boldsymbol{C}_{\boldsymbol{2,1}}\boldsymbol{/}\boldsymbol{C}_{\boldsymbol{2,2}}\boldsymbol{=1.00}$**), (b)** $\boldsymbol{C}_{\boldsymbol{2,1}}\boldsymbol{/}\boldsymbol{C}_{\boldsymbol{2,2}}\boldsymbol{=0.64}$**, and (c)** $\boldsymbol{C}_{\boldsymbol{2,1}}\boldsymbol{/}\boldsymbol{C}_{\boldsymbol{2,2}}\boldsymbol{=0.47}$**. In each case the peak** $\boldsymbol{\sigma}_{\boldsymbol{vm}}$ **and** $T_{t}$ **are coincident, however their location follows the tighter radius of curvature at the aneurysm neck, and** $\boldsymbol{C}_{\boldsymbol{2,1}}$**=7.**

*Aneurysm Ellipticity* $(W_{1}/W_{2})$*:*

Next, we investigate aneurysm ellipticity and observe that $\sigma_{vm}$ and $T_{t}$ always coincide. In our baseline axisymmetric simulation (W_1_/W_2_=1.00) $\sigma_{vm}$ and $T_{t}$ are coincident and identical circumferentially at the aneurysm neck, as ellipticity increases (W_1_/W_2_=[0.90, 0.80]), both $\sigma_{vm}$ and $T_{t}$ decrease to a minimum at the minor axis width (**Figure S2**).


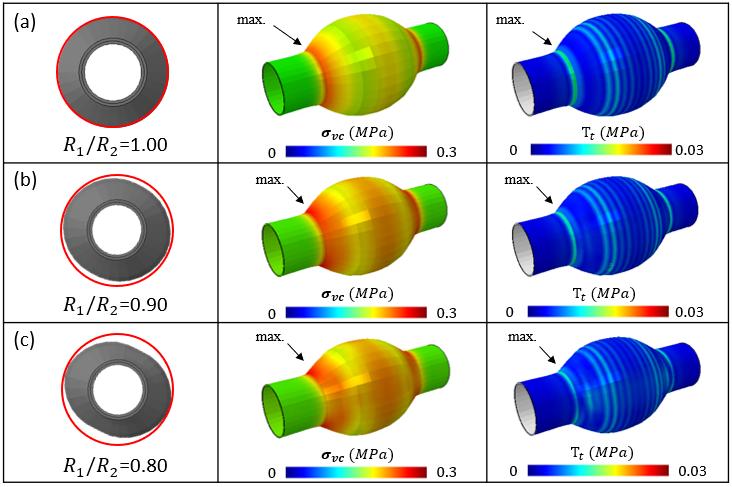


**Figure S2: Effect of ellipticity (**$\boldsymbol{W}_{\boldsymbol{1}}\boldsymbol{/}\boldsymbol{W}_{\boldsymbol{2}}$**) on the peak** $\boldsymbol{\sigma}_{\boldsymbol{vm}}$**/**$T_{t}$ **relationship. (a) Baseline geometry (**$\boldsymbol{W}_{\boldsymbol{1}}\boldsymbol{/}\boldsymbol{W}_{\boldsymbol{2}}\boldsymbol{=1.00}$**), (b)** $\boldsymbol{W}_{\boldsymbol{1}}\boldsymbol{/}\boldsymbol{W}_{\boldsymbol{2}}\boldsymbol{=0.90}$**, and (c)** $\boldsymbol{W}_{\boldsymbol{1}}\boldsymbol{/}\boldsymbol{W}_{\boldsymbol{2}}\boldsymbol{=0.80}$**. In each case the peak** $\boldsymbol{\sigma}_{\boldsymbol{vm}}$ **and** $T_{t}$ **are coincident, however both decrease to a minimum at the minor axis width, and** $\boldsymbol{L/R}$**=2.5.**

Finally, we investigate the effect of material anisotropy on the peak $\sigma_{vm}$/$T_{t}$ relationship. By employing a bilinear type constitutive model that captures the strain-stiffening of collagen fibers we can capture the stress-strain behaviour of AAA tissue (Vande Geest et al., 2006). Applying these fitted parameters to the pressure inflation of the baseline AAA geometry, we observe that the peak $\sigma_{vm}$ resides at the aneurysm belly in the anisotropic model whereas in the isotropic model it resides at the aneurysm neck. In both the isotropic and anisotropic case however the peak $\sigma_{vm}$ and $T_{t}$ are coincident (**Figure S3**).


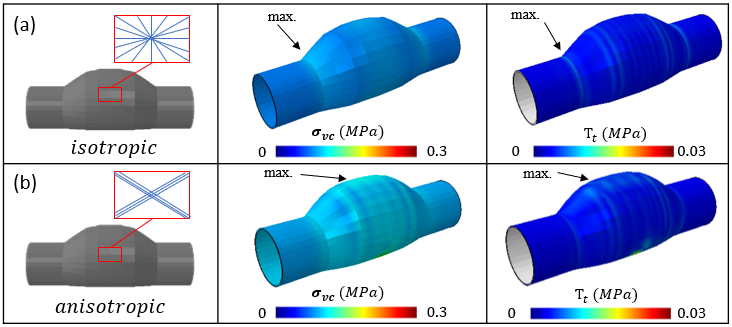


**Figure S3: Effect of material anisotropy on the peak** $\boldsymbol{\sigma}_{\boldsymbol{vm}}$**/**$T_{t}$ **relationship. (a) Baseline geometry (isotropic), (b) Baseline geometry (anisotropic). In the case of isotropic model, the peak** $\boldsymbol{\sigma}_{\boldsymbol{vm}}$ **and** $T_{t}$ **coincide at the aneurysm neck, however in the anisotropic model they coincide at the aneurysm belly.**

### Conditions Required to Cause I-M Delamination Prior to A-M Delamination

For Patient A, A-M delamination is computed prior to I-M delamination (initial delamination is computed at 132mmHg in the A-M interface as opposed to 138mmHg at the I-M interface). However, it has been observed through CT imaging that dissections primarily occur at the I-M interface in aneurysms (Hyodoh, 1996). Additionally, it has been reported that the intima is a mechanically significant layer of considerable thickness and stiffness for human aged arteries, in contrast to healthy cardiovascular tissue where the intima is relatively thin (Holzapfel, 2005, 2007). Therefore, considering Patient A, an investigation into the conditions which lead to A-M delamination prior to I-M delamination is carried out. Specifically, both the intima thickness and stiffness are doubled. Results are summarised in **Table S2**. As reported above, initial delamination occurs at the lateral right (LR) location of the A-M interface prior to I-M delamination at a lumen pressure of 132mmHg for an intima thickness of 0.17mm and $E_{\mathrm{intima}}$=2.9MPa. I-M delamination is computed at a higher pressure at the lateral right (LR) and anterior bulge (ANT) locations at 138mmHg and 168mmHg, respectively. An increase in intima stiffness to $E_{\mathrm{intima}}$=5.8MPa also results in an initial A-M tear computed at 131mmHg in the lateral right aneurysm location. Interestingly, the increase in intima stiffness also increases the pressure at which I-M delamination is computed in the lateral right location (146mmHg), relative to the less stiff intima. Additionally, for the increased intima stiffness, no delamination is computed in the anterior region of the aneurysm. It is evident in **Table S2** that an increase in intima thickness (0.34mm) significantly reduces the pressure at which I-M delamination are computed, regardless of intima stiffness. Specifically, I-M delamination is computed prior to A-M delamination for $E_{\mathrm{intima}}$=2.9MPa while interlayer delamination are computed at approximately the same pressure for $E_{\mathrm{intima}}$=5.8MPa. Therefore, it is evident that accurate intima stiffness and thickness measurements are crucial in order to accurately predict initial AAA dissection locations.

**Table S2: Table summarising the influence of intima thickness and intima stiffness (**$\boldsymbol{E}_{\boldsymbol{intima}}$**) on initial delamination pressure and location for Patient A. A-M and I-M refer to the adventitia-media and intima-media interfaces, respectively. Areas LR, LL and ANT refer to lateral right, lateral left, and anterior AAA locations, respectively. All pressures are given in mmHg.**

| **Intima thickness (mm)** | **E _intima_ (MPa)** | **Pressure (mmHg)** | **Interface** | **Location** |
| --- | --- | --- | --- | --- |
| 0.17 | 2.9 | 132 | A-M | LR |
| 0.17 | 2.9 | 138 | I-M | LR |
| 0.17 | 2.9 | 168 | I-M | ANT |
| 0.17 | 5.8 | 131 | A-M | LR |
| 0.17 | 5.8 | 146 | I-M | LR |
| 0.34 | 2.9 | 120 | A-M | LR |
| 0.34 | 2.9 | 115 | I-M | LR |
| 0.34 | 2.9 | 111 | I-M | LL |
| 0.34 | 5.8 | 117 | A-M | LR |
| 0.34 | 5.8 | 118 | I-M | LR |


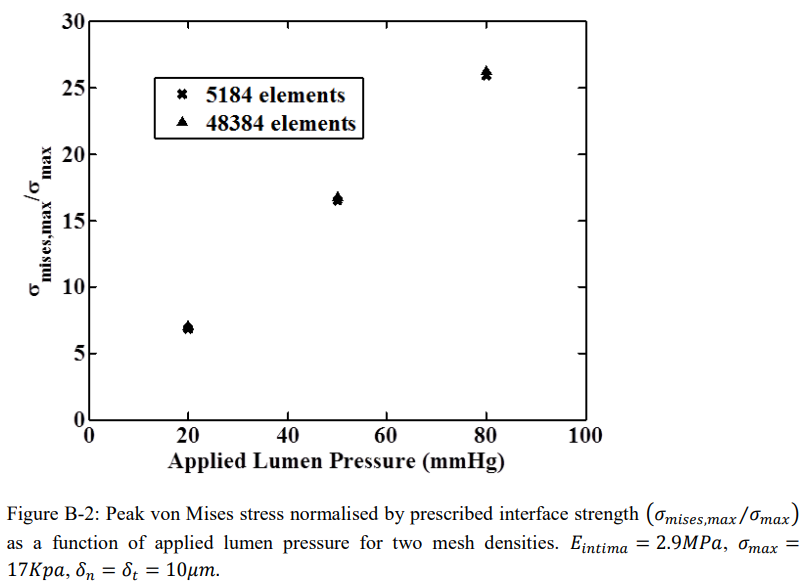


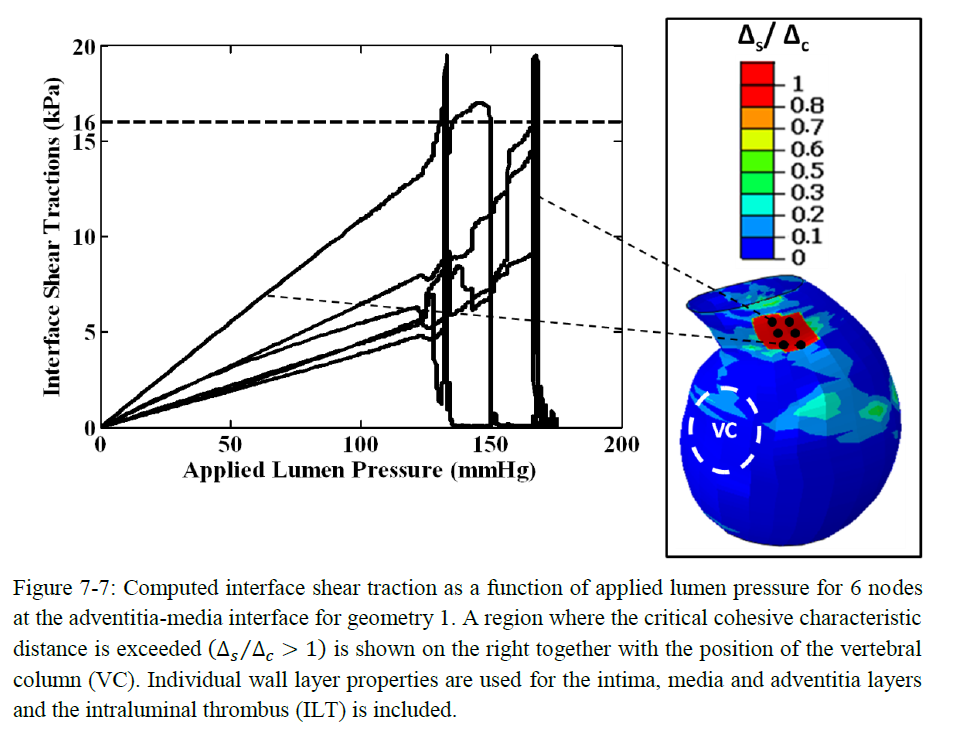


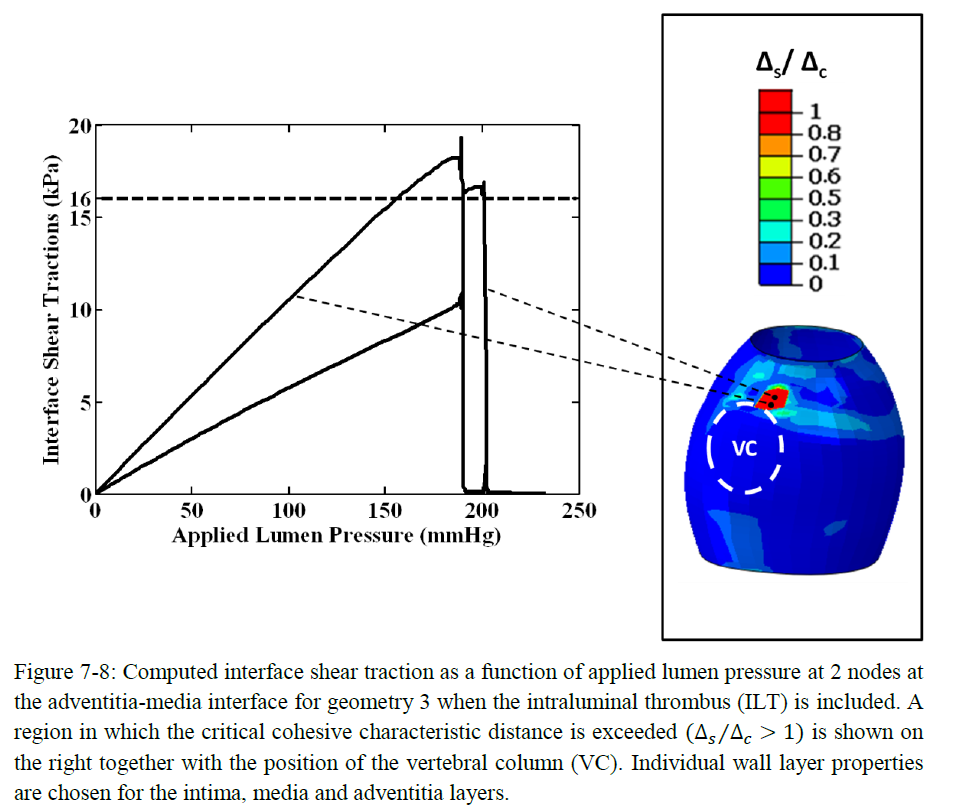

Supplement: Supplementary file 1 — Supplementary file1 (DOCX 892 kb) [file 13239_2024_740_MOESM1_ESM.docx]
